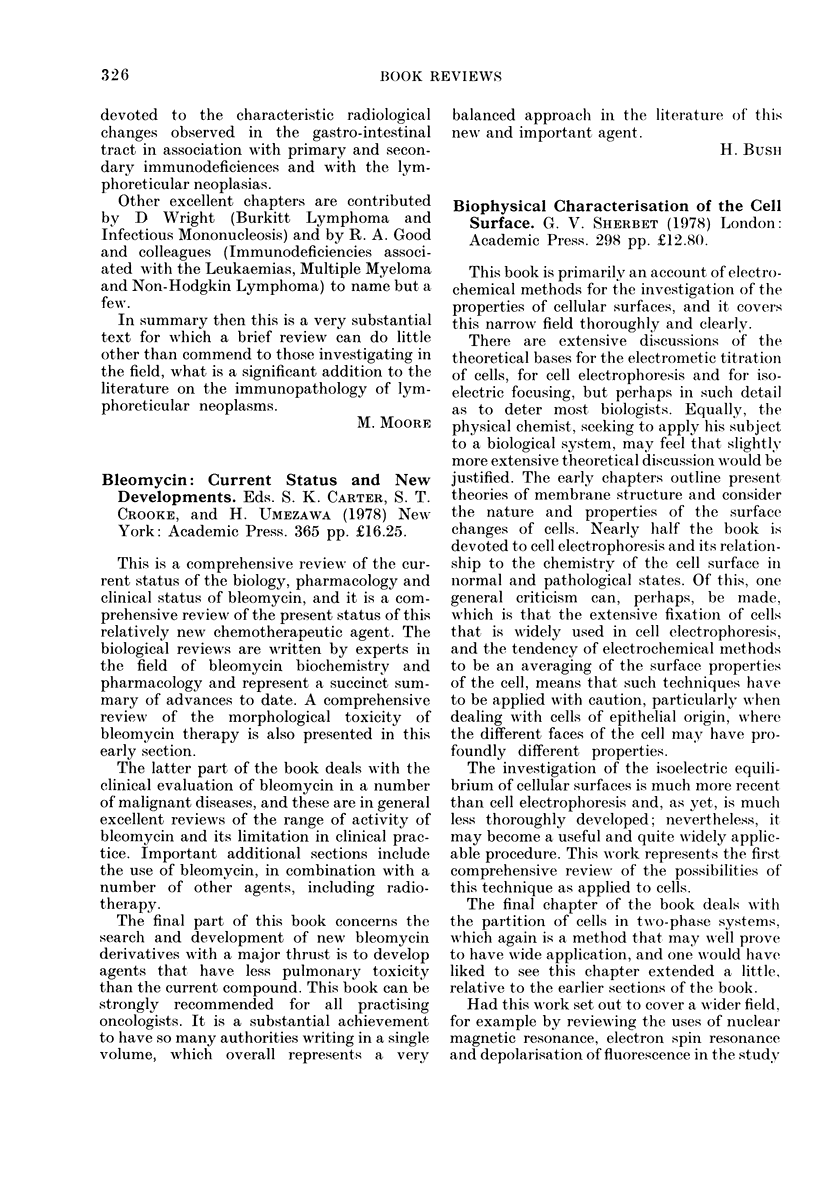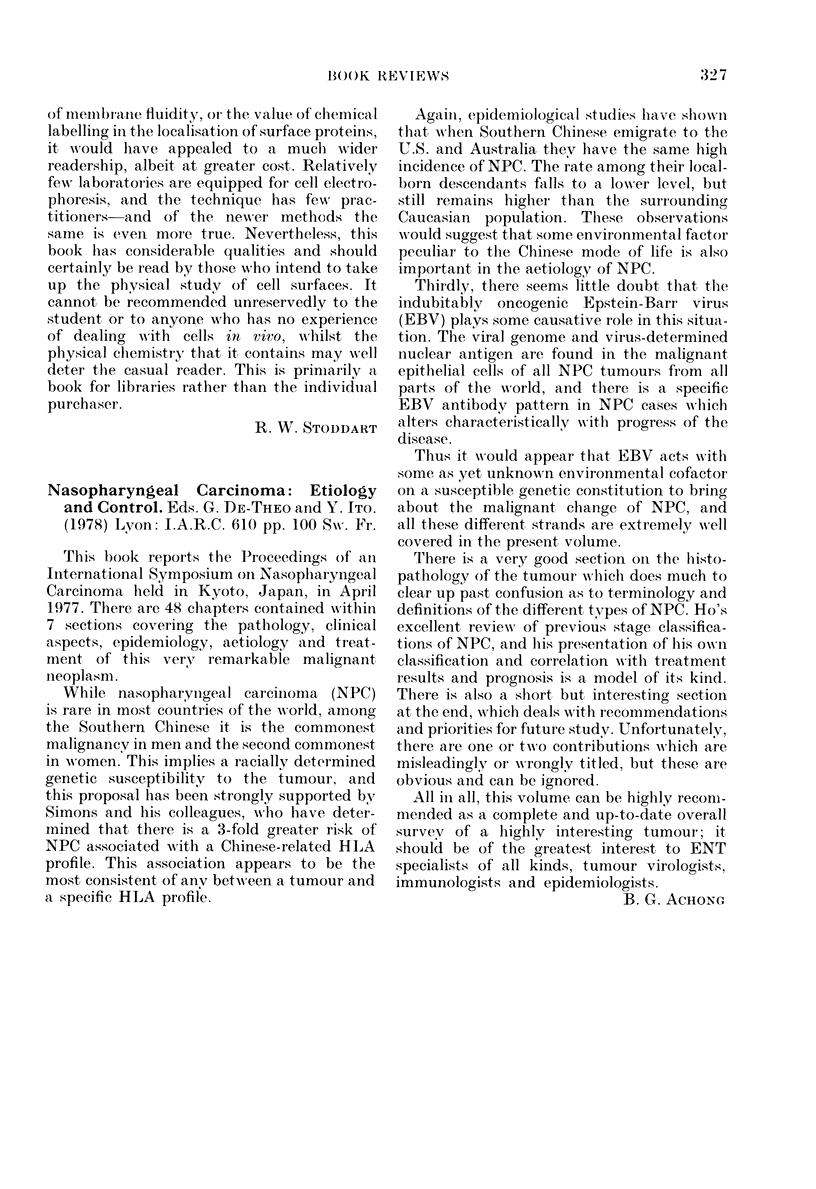# Biophysical Characterisation of the Cell Surface

**Published:** 1979-08

**Authors:** R. W. Stoddart


**Biophysical Characterisation of the Cell Surface.** G. V. SHERBET (1978) London: Academic Press. 298 pp. £12.80.

This book is primarily an account of electrochemical methods for the investigation of the properties of cellular surfaces, and it covers this narrow field thoroughly and clearly.

There are extensive discussions of the theoretical bases for the electrometic titration of cells, for cell electrophoresis and for isoelectric focusing, but perhaps in such detail as to deter most biologists. Equally, the physical chemist, seeking to apply his subject to a biological system, may feel that slightly more extensive theoretical discussion would be justified. The early chapters outline present theories of membrane structure and consider the nature and properties of the surface changes of cells. Nearly half the book is devoted to cell electrophoresis and its relationship to the chemistry of the cell surface in normal and pathological states. Of this, one general criticism can, perhaps, be made, which is that the extensive fixation of cells that is widely used in cell electrophoresis, and the tendency of electrochemical methods to be an averaging of the surface properties of the cell, means that such techniques have to be applied with caution, particularly when dealing with cells of epithelial origin, where the different faces of the cell may have profoundly different properties.

The investigation of the isoelectric equilibrium of cellular surfaces is much more recent than cell electrophoresis and, as yet, is much less thoroughly developed; nevertheless, it may become a useful and quite widely applicable procedure. This work represents the first comprehensive review of the possibilities of this technique as applied to cells.

The final chapter of the book deals with the partition of cells in two-phase systems, which again is a method that may well prove to have wide application, and one would have liked to see this chapter extended a little, relative to the earlier sections of the book.

Had this work set out to cover a wider field, for example by reviewing the uses of nuclear magnetic resonance, electron spin resonance and depolarisation of fluorescence in the study of membrane fluidity, or the value of chemical labelling in the localisation of surface proteins, it would have appealed to a much wider readership, albeit at greater cost. Relatively few laboratories are equipped for cell electrophoresis, and the technique has few practitioners—and of the newer methods the same is even more true. Nevertheless, this book has considerable qualities and should certainly be read by those who intend to take up the physical study of cell surfaces. It cannot be recommended unreservedly to the student or to anyone who has no experience of dealing with cells *in vivo*, whilst the physical chemistry that it contains may well deter the casual reader. This is primarily a book for libraries rather than the individual purchaser.

R. W. STODDART